# Metabolic Variation Dictates Cardiac Pathogenesis in Patients With Tetralogy of Fallot

**DOI:** 10.3389/fped.2021.819195

**Published:** 2022-01-31

**Authors:** Jianyang Liu, Shuxin Kong, Shubo Song, Haoju Dong, Zhidong Zhang, Taibing Fan

**Affiliations:** ^1^Department of Vascular Surgery, Zhengzhou University People's Hospital, Henan Provincial People's Hospital, Fuwai Central China Cardiovascular Hospital, Zhengzhou, China; ^2^Department of Children's Heart Center, Zhengzhou University People's Hospital, Henan Provincial People's Hospital, Fuwai Central China Cardiovascular Hospital, Zhengzhou, China; ^3^Department of Breast Surgery, Zhengzhou University People's Hospital, Henan Provincial People's Hospital, Zhengzhou, China

**Keywords:** tetralogy of Fallot, metabolic reprogramming, metabonomic analysis, UPLC–MS/MS, butanoate metabolism

## Abstract

**Background:**

Herein, we aimed to analyze cardiac metabolic reprogramming in patients with tetralogy of Fallot (ToF).

**Methods:**

Cardiac metabolic reprogramming was analyzed through comprehensive bioinformatics analysis, which included gene set enrichment, gene set variation, and consensus clustering analyses, so as to assess changes in metabolic pathways. In addition, full-spectrum metabolomics analysis was performed using right atrial biopsy samples obtained from patients with ToF and atrial septal defect (ASD) before cardiopulmonary bypass; ultrahigh performance liquid chromatography–tandem mass spectrometry (UPLC–MS/MS) was used to construct a metabolic map of cardiac metabolic reprogramming in cyanotic congenital heart disease.

**Results:**

The metabolic maps of carbohydrate metabolic process and heme metabolism were significantly activated, while bile acid metabolism, lipid droplet, and lipid binding were primarily restrained in ToF samples as compared with that in ASD samples. The reprogramming of butanoate metabolism was identified basing on the UPLC–MS/MS detection and analysis in myocardial hypoxia damage in cyanotic heart disease. Finally, the butanoate metabolism–related hub regulators ALDH5A1 and EHHADH were identified and they were significantly downregulated in ToF samples.

**Conclusions:**

The metabolic network of butanoate metabolism involved ALDH5A1 and EHHADH, which could contribute to myocardial tissue damage in cyanotic congenital heart of ToF. Our results provide further insights into the mechanisms underlying metabolic reprogramming in cyanotic congenital heart disease and could lead to the identification of potential therapeutic targets.

## Introduction

Tetralogy of Fallot (ToF) is the most common cyanotic congenital heart disease, with an overall incidence of 0.35%, accounting for ~3.5–5% of all congenital heart diseases (1). ToF may arise due to the abnormal development of the conotruncus during the fetal period, with deviation occurring between the anterior and cephalad parts of the heart and the infundibular septum, resulting in malalignment between the right ventricular inlet and outlet and the ventricular septum. It mainly manifests as pulmonary stenosis, ventricular septal defect, overriding aorta, and right ventricular hypertrophy. Approximately 50% of children present with double outlet right ventricle and 20% with coronary artery anomalies (2, 3). Children with ToF develop chronic systemic hypoxia, arrhythmia, pneumonia, and other complications after birth. The natural prognosis of ToF is poor. In the absence of timely treatment, as high as up to 90% children do not survive to adulthood, succumbing mainly to chronic hypoxia-induced secondary myocardial hypertrophy and heart failure (3). At present, the vast majority of ToF cases can be corrected owing to improved surgical repair techniques, but still, ~5% of children are prone to sudden death or poor long-term outcomes due to postoperative complications, such as arrhythmias, leading to heavy economic, and mental burdens on the families and society (2).

Cardiac development is a multifaceted process, involving a series of complex and specific signaling pathways and transcriptional cascades (4). The major gene alterations involved in ToF as the most common conotruncal anomaly include 22q11.2 deletion syndrome and alterations in genes such as NKX2.5, TBX20, GATA4, JAG1, FOXC2, and TBX1 (4). Both environmental and genetic factors play a role in ToF development (3, 5, 6). Environmental factors mediate its occurrence mainly through gene mutations and epigenetic alterations. In children with ToF, long-term chronic cardiac hypoxia results in metabolic microenvironment changes and metabolic reprogramming in the myocardial tissue (7, 8). Naviaux et al. found that in response to myocardial ischemic injury, cells activate a cascade of pathological processes, such as energy metabolism, mitochondrial dysfunction, and fibrosis remodeling. Besides, in response to cell danger response that persists abnormally, the myocardial tissue metabolic microenvironment causes reprogramming, resulting in an induced mitochondrial dysfunction (9). Mattson et al. suggested that the cardiac T cell oxidation–reduction (redox) system plays a key role in pathological conditions, including cardiac hypertension, by regulating metabolic processes (10). Nguyen et al. illustrated that cardiac lipid metabolism dysfunction is directly involved in ATP production, mitochondrial dysfunction, and cell death, and this occurs *via* the regulation of mitochondrial and cellular macromolecular content (11). Nevertheless, the mechanism underlying cardiac metabolic reprogramming in ToF remains unclear.

## Materials and Methods

### Transcriptome Microarray Analysis

**Figure 1:**
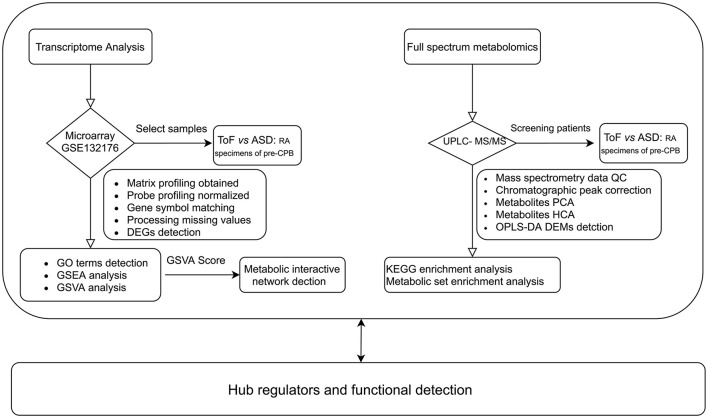
Workflow of metabolic reprogramming analysis.

The gene expression profile of ToF was downloaded from the Gene Expression Omnibus (GEO) database GSE132176 (https://www.ncbi.nlm.nih.gov/geo/) (12). Ten ToF and 10 atrial septal defect (ASD) right atrium (RA) specimens collected from patients before cardiopulmonary bypass (CPB) were chosen from GSE132176 (13). Subsequently, CEL raw data were normalized with the “affyPLM” and “affy” algorithms. The analytical process included the following steps: (1) matrix file obtained from fluorescent images based on the permuted language model, (2) probe profiling normalized using the robust multiarray average method (14), (3) gene symbol matching achieved based on the GPL13158 Affymetrix HT HG-U133+ PM Array Plate, (4) missing values filled in via the k-nearest neighbor algorithm, and (5) differentially expressed genes (DEGs) detected by linear models for microarray data analysis (15). Benjamini–Hochberg-adjusted *p* ≤ 0.05 and log_2_|fold change (FC)| ≥ 1.0 were the criteria to identify DEGs. The analysis flowchart of this study is shown in Figure 1.

### Metabolic Pathway and Network Interaction Analyses

The ToF and ASD RA specimens collected from patients before CPB were subjected to gene set enrichment analysis (GSEA; http://software.broadinstitute.org/gsea/index.jsp) with the “clusterProfiler” and “AnnotationHub” algorithms (16). The number of genes in the minimum gene set was 10 and that in the largest gene set was 500. ToF-related DEGs were then subjected to gene ontology (GO) term enrichment analysis. Benjamini–Hochberg-corrected *p* < 0.05 and number of permutations = 1,000 were the criteria to identify significantly enriched pathways. Of the pathway score, the gene list of hallmark: heme metabolism, GOBP: carbohydrate metabolic process, hallmark: fatty acid metabolism, GOCC: lipid droplet, hallmark: xenobiotic metabolism, hallmark: bile acid metabolism, GOMF: lipid binding, and GOBP: response to xenobiotic stimulus were obtained from the GSEA database. In addition, the score of metabolic related maps was calculated using the “GSVA” and “msigdbr” R packages (17). Finally, partial correlation analysis was performed to analyze the normalized pathway score to detect interaction relationships.

### Mass Spectrometry (MS)–Based Metabolomic Profiling

We selected 12 samples and detected 1,402 metabolites using widely targeted metabolomics. Metabolomic differences between samples were compared by performing ultrahigh performance liquid chromatography (UPLC; ExionLC AD, USA)–tandem mass spectrometry (MS/MS; QTRAP, USA), constructing a database, and employing multivariate statistical analysis (18). Metabolomics data were acquired through experimental design, sample collection and processing, and metabolite extraction and measurement, followed by metabolite identification and quality control analysis of sample data, screening for metabolites showing differences, and functional prediction and analysis of sample metabolites.

Analyst 1.6.3 (SCIEX) was used to process MS data (19). Pooled samples were used for quality control. Total ion current (TIC) and multiple reaction monitoring chromatograms were plotted. Using a triple quadrupole mass spectrometer, the characteristic ion of each substance was screened, and signal intensity (counts per second) of the characteristic ion was measured in a detector. MultiQuant was used for chromatographic peak integration and correction using mass spectrometer output files (20). Peak area represented the relative content of a specific substance. All chromatographic peak area integration data were exported and saved. To compare the content of each detected metabolite in different samples, the chromatographic peak was corrected based on the retention time and peak type of a metabolite, which ensured qualification and quantification accuracy.

### Principal Component Analysis (PCA)

PCA was performed to investigate overall metabolic differences among all groups of samples and variations within groups (21). PCA helped us determine whether there was separation in the metabolome among groups, which in turn indicated if differences were present in the metabolome.

### Hierarchical Cluster Analysis (HCA)

HCA clusters individuals or objects based on their characteristics, with individuals or objects within one cluster being as homogeneous as possible, with clusters being as heterogeneous as possible (22). Metabolite content data were normalized by the unit variance scaling method. HCA was performed to analyze the accumulation pattern of metabolites in different samples.

### Orthogonal Partial Least Squares Discriminant Analysis (OPLS-DA)

To avoid missing the information pertaining to differentially expressed metabolites (DEMs) between low-correlation samples in PCA, we performed PLS-DA, a supervised multivariate statistical method (23). This method extracts the components of independent and dependent variables separately and calculates the correlation between components. In comparison with PCA, PLS-DA maximizes differences between groups, facilitating the identification of DEMs. OPLS-DA, which combines orthogonal signal correction and PLS-DA, can decompose the matrix information of independent variables into dependent variable–related and dependent variable–unrelated information, and remove unrelated differences to screen for differential variables, considerably enriching differential analysis results (23). Variable importance in projection (VIP) score, which was derived from the OPLS-DA model, was used to preliminarily identify DEMs; in addition, p or FC in univariate analysis was used for further screening. VIP ≥1.0 and log_2_|FC| ≥1.0 were used as the criteria to identify DEMs.

### Functional Annotation and Enrichment Analysis of DEMs

Based on Kyoto Encyclopedia of Genes and Genomes (KEGG, https://www.genome.jp/kegg/), we studied the interactions and pathways of DEMs, mainly including the possible metabolic pathways of carbohydrate, nucleotide, and amino acid metabolism and organic compound biodegradation; moreover, comprehensive annotation of enzymes for each reaction step was performed (24). A *p* < 0.05 for pathway enrichment was considered to be statistically significant.

### Metabolite Set Enrichment Analysis (MSEA)

Hypergeometric distribution-based traditional enrichment analysis is mainly applied to significantly up- or downregulated DEMs, and some metabolites showing no significant differences but having important biological significance are thus overlooked. Basing on the MetaboAnalyst (https://www.metaboanalyst.ca/) database, we performed MSEA, which identifies a series of metabolic sets without specifying the threshold for DEMs, and enriches metabolomics data into these metabolic sets so as to identify those with significant differences (25). A *p* < 0.05 indicated statistical significance.

### Hub Regulators and Functional Detection

The gene list of most significant metabolic pathways was downloaded from the GSEA database. Subsequently, differential expression levels of these genes were further analyzed. The validation dataset GSE169214 was downloaded in response to the hypoxic or normoxic environment of the cardiac tissue in the mouse model for cyanotic congenital heart disease. The “Oligo” algorithm was used for raw data preprocessing (26). Consequently, the online version of ToppGene Suite (https://toppgene.cchmc.org/) was used for hub gene functional enrichment (27). Terms with *p* < 0.05 were considered to be significantly enriched.

## Results

### Detecting DEGs and Enriched Pathways

**Figure 2:**
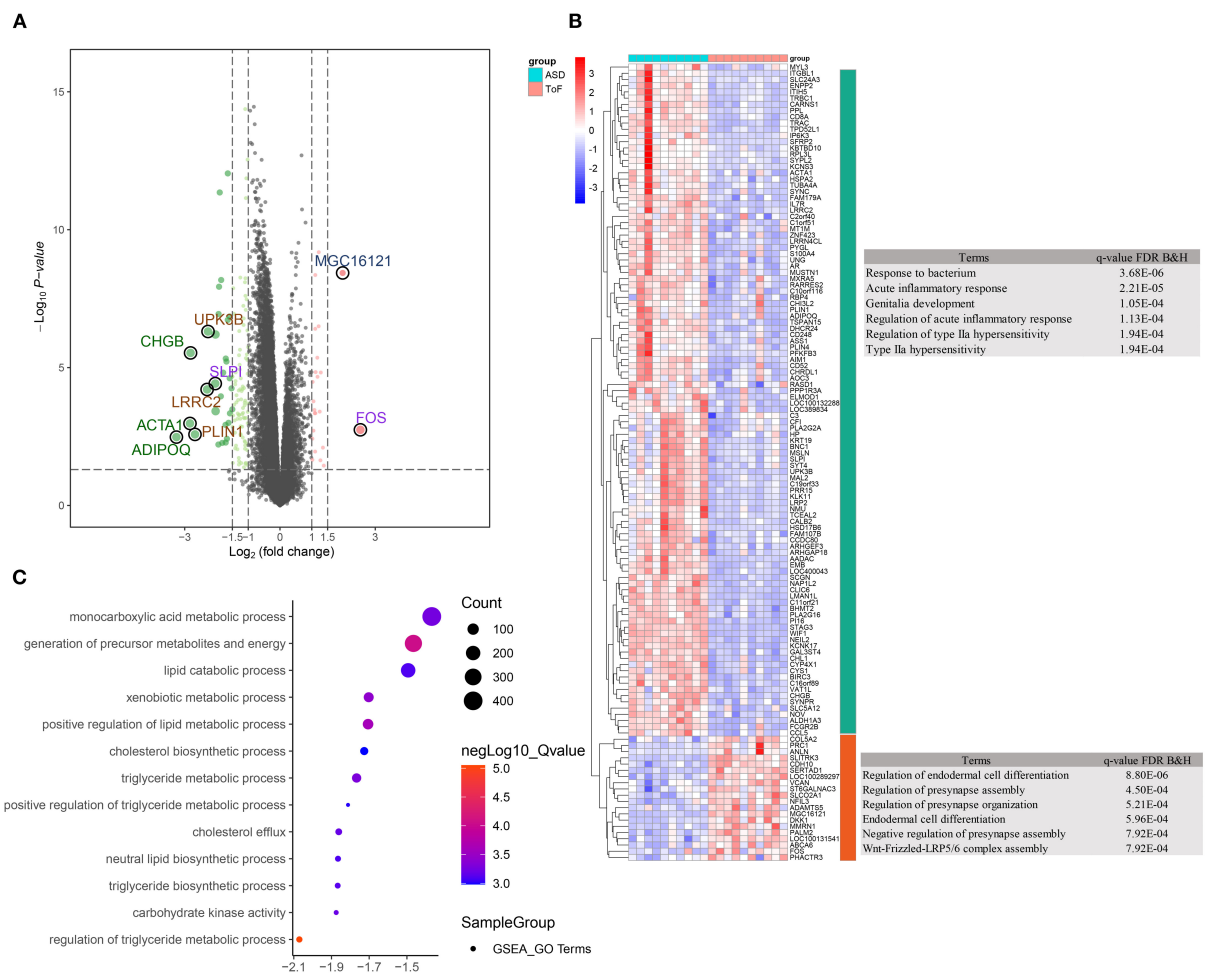
Differentially expressed gene (DEG) identification and functional enrichment analysis. **(A)** Volcano plot showing DEG distribution. **(B)** Clustering heatmap and biological analysis presenting the biological function of up- and downregulated DEGs. **(C)** Biological function terms based on gene ontology were calculated by the GSEA algorithm in response to the comparison between ToF and ASD RA specimens.

Overall, 128 significant DEGs were detected, which included 108 down- and 20 upregulated DEGs, in ASD RA samples compared with ToF samples. A volcano plot and expression heatmap are shown in Figure 2A and Supplementary Table 1, respectively. The downregulated DEGs significantly correlated with the maps of response to bacterium (*p* = 3.68E−06, gene count = 15), acute inflammatory response (*p* = 2.21E−05, gene count = 6), and genitalia development (*p* = 1.05E−04, gene count = 4), in addition to regulation of endodermal cell differentiation (*p* = 8.80E−06, gene count = 2), regulation of presynapse assembly (*p* = 4.50E−04, gene count = 2), and regulation of presynapse organization (*p* = 5.21E−04, gene count = 2) (Figure 2B; Supplementary Table 1). Furthermore, GSEA results showed that the metabolic categories of regulation of triglyceride metabolic process (size = 26, enrichment score = −0.69, NES = −2.07, adjusted *p* = 0.003), carbohydrate kinase activity (size = 18, enrichment score = −0.68, NES = −1.88, adjusted *p* = 0.037), triglyceride biosynthetic process (size = 23, enrichment score = −0.64, NES = −1.87, adjusted *p* = 0.041), neutral lipid biosynthetic process (size = 24, enrichment score = −0.64, NES = −1.87, adjusted *p* = 0.042), and cholesterol efflux (size = 32, enrichment score = −0.59, NES = −1.86, adjusted *p* = 0.038) were significantly associated with chronic hypoxia-induced ToF-related myocardial pathogenesis (Figure 2C; Supplementary Table 2).

### Metabolic Pathway and Network Interaction Analyses

**Figure 3:**
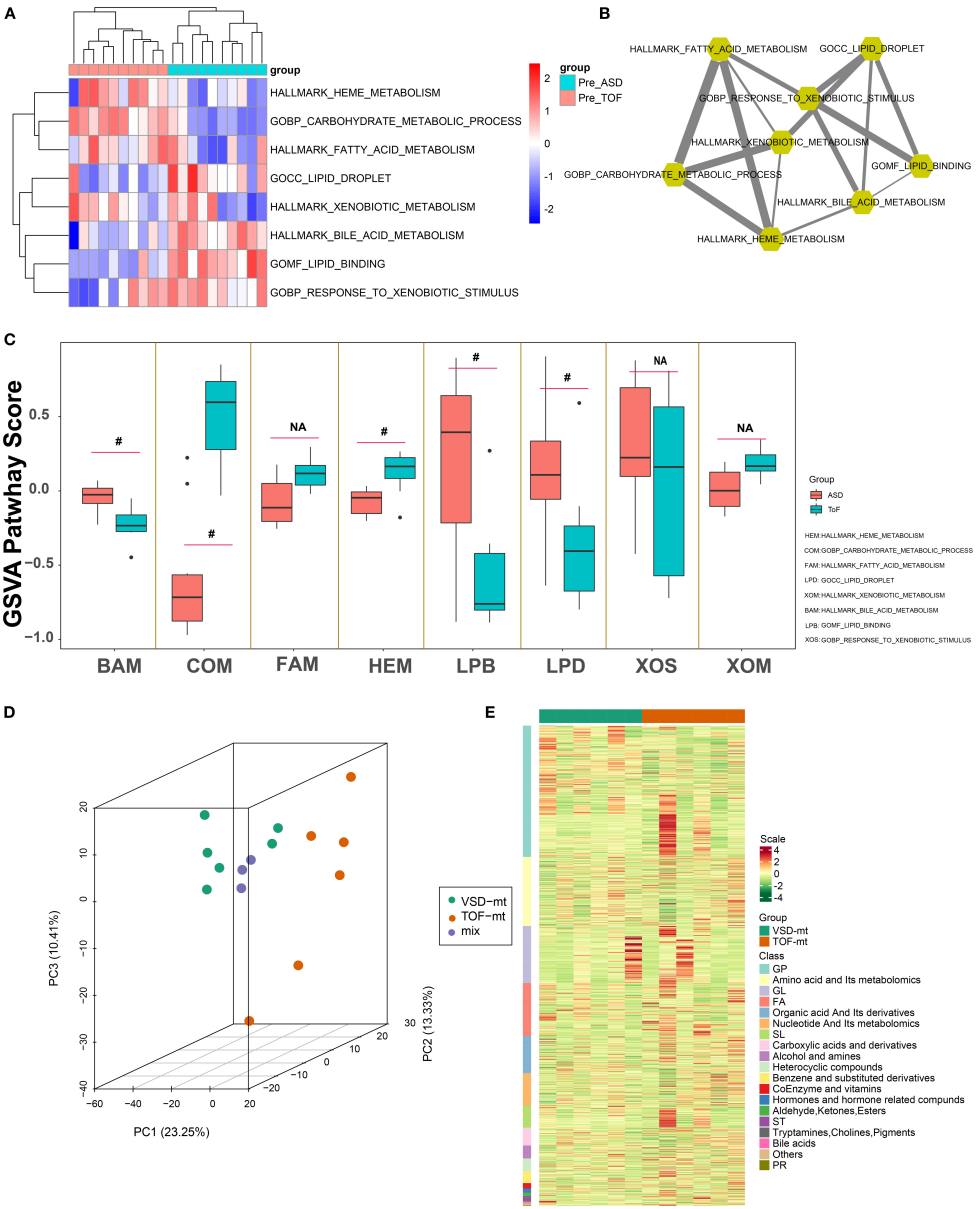
Metabolic pathway detection and interaction network construction. **(A)** Clustering heatmap showing GSVA-quantized pathway score of metabolic maps. **(B)** Metabolic map interaction analysis was identified via weighted partial correlation analysis. **(C)** Differential analysis of metabolic pathways of GSVA score in Figure 2C. **(D)** Three-dimensional principal component analysis, showing right atrial biopsy sample distribution of ToF and ASD patients before cardiopulmonary bypass. **(E)** Clustering heatmap depicting the standard quantification of metabolites in ToF and ASD RA specimens. ^#^*p* < 0.05.

Gene set variation analysis (Figure 3A; Supplementary Table 3) revealed that ToF and ASD RA samples showed a significant difference in the metabolism-related pathways of lipid droplet (*p* = 0.011), lipid binding (*p* = 0.0037), carbohydrate metabolic process (*p* = 3.23E−06), and heme metabolism (*p* = 0.011). In addition, partial correlation analysis demonstrated a direct association among these metabolism-related terms (Figure 3B). These results suggested that metabolic reprogramming changes were related to myocardial pathogenesis in ToF-related chronic hypoxia.

### Qualitative and Quantitative Analysis of Metabolites

Overall, 12 male patients (6 with ToF and 6 with ASD) were included. All were diagnosed based on cardiac color Doppler ultrasound or cardiac CT examination. Those with severe pulmonary stenosis, severe heart failure, and other complex malformations were excluded. Herein, the age of ToF ranged from 13 to 18 months and that of ASD ranged from 15 to 19 months. The right ventricle end diastolic volume in patients with ToF ranged from 55 to 185 ml/m^2^. The pro-brain natriuretic peptide level in patients with ToF and ASD ranged from 3.6 to 25.1 and 1.5 to 14.6 pmol/L (within limit), respectively. Of the metabolite analysis, the superposition of the TIC of the MS detection of the quality control sample indicated that the curve overlap of the TIC of the metabolite detection was high, and the MS signal for sample detection was stable. PCA results showed the separation trend of the metabolome between groups, suggesting the presence of significant differences in the metabolome between sample groups (Figure 3C). Furthermore, after normalization of the metabolite expression profile, sample clustering analysis was applied to comprehend the cluster tree distribution of metabolites among ToF and ASD RA samples (Figure 3D).

### PLS-DA

**Figure 4:**
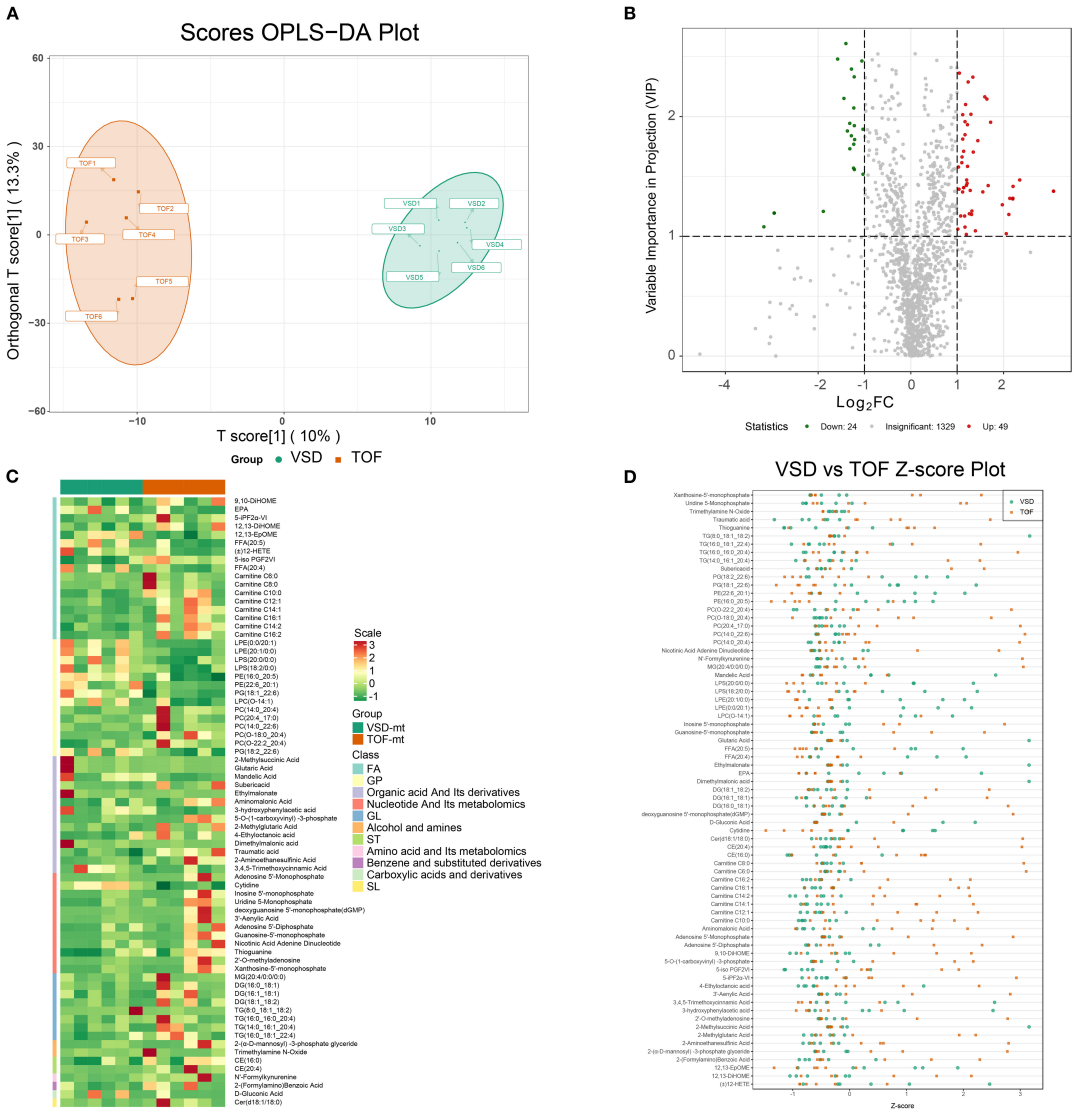
Detection of differentially expressed metabolites in ToF and ASD RA specimens via the UPLC–MS/MS spectra. **(A)** T-score of OPLS-DA showing the distribution of ToF and ASD specimens. **(B)** Volcano plot showing the distribution of differentially expressed metabolites. **(C)** Differential expression of metabolites in ToF and ASD specimens. **(D)** Scatter diagram of Figure 3D showing the standardized Z-score for each sample.

To mine the weak-correlated features in these metabolites, the widely used method based on the OPLS-DA for metabolite detection is carried out (Figure 4A).

### Detecting DEMs

Based on VIP scores derived from the OPLS-DA model, DEMs associated with ToF-related myocardial pathogenesis were identified. In total, 73 metabolites showed significant differential expression, 49 of which were overexpressed in ToF and 24 in ASD (Figure 4B). Figure 4C shows the DEM clustering tree and heatmap for class II metabolites (Supplementary Table 4). Of the clustering results, the components were classified as nucleotide and its metabolomics, glycerol phospholipids, and glycerol lipids showing significant differences. To avoid interference of abnormal distributed metabolomics data, we applied standardized Z-score to analyze and visualize differences in metabolomics data (Figure 4D).

Herein TG (8:0_18:1_18:2) (VIP score = 1.08; log_2_FC = −3.17), 2-methylsuccinic acid (VIP score = 1.19; log_2_FC = −2.95), glutaric acid (VIP score = 1.19; log_2_FC = −2.95), ethylmalonate (VIP score = 1.19; log_2_FC = −2.94), and dimethylmalonic acid (VIP score = 1.19; log_2_FC = −2.94) were primarily downregulated, while adenosine-5′-monophosphate (VIP score = 1.38; log_2_FC = 3.08), 2′-deoxyguanosine-5′-monophosphate (VIP score = 1.38; log_2_FC = 3.08), guanosine-5′-monophosphate (VIP score = 1.47; log_2_FC = 2.35), xanthosine-5′-monophosphate (VIP score = 1.42; log_2_FC = 2.21), and 3′-aenylic acid (VIP score = 1.32; log_2_FC = 2.20) were overexpressed in ASD samples (Figure 4D; Supplementary Table 4).

### Functional Analysis of DEMs

**Figure 5:**
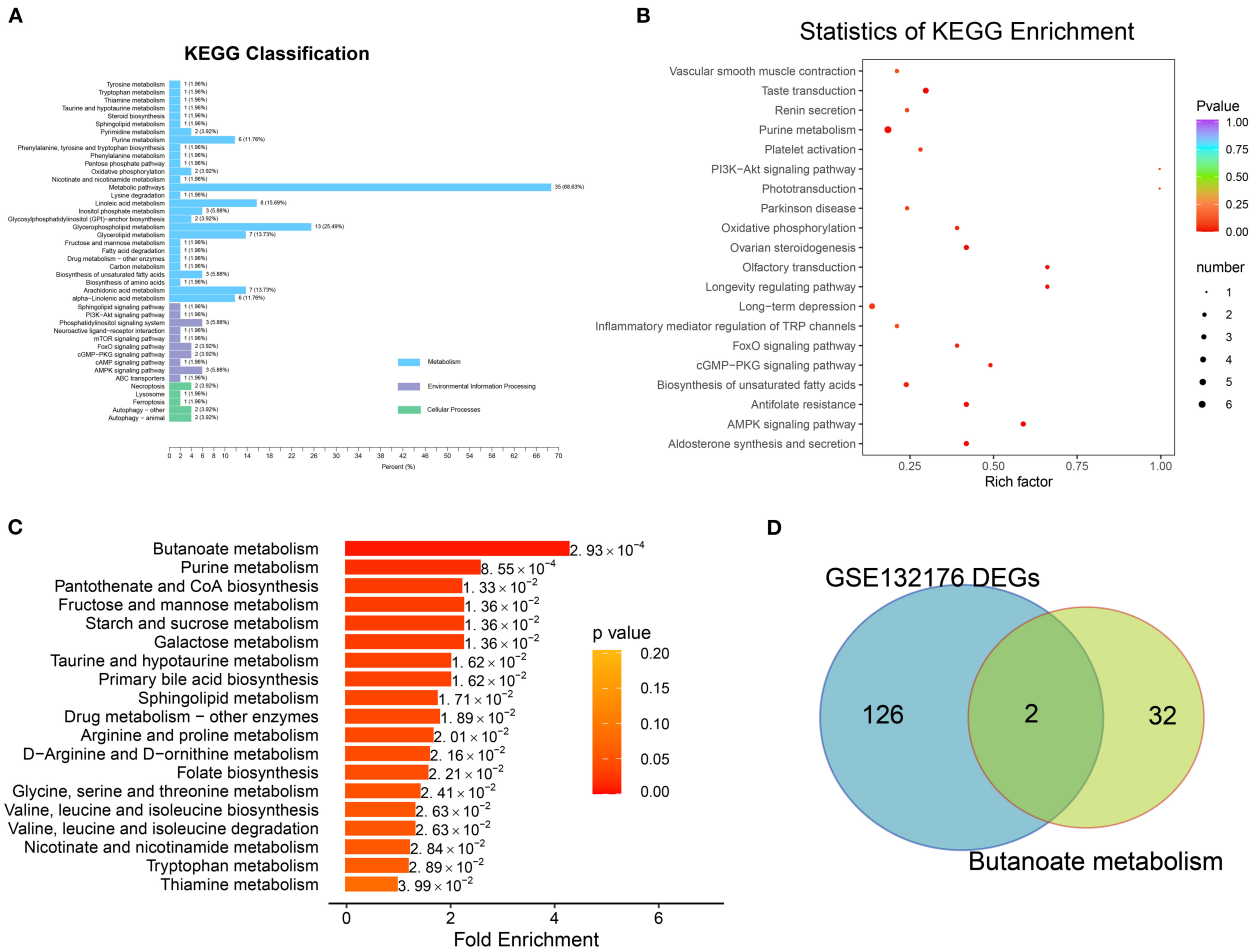
Pathway enrichment analysis with differentially expressed metabolites. **(A)** The Kyoto Encyclopedia of Genes and Genomes (KEGG) pathway analysis involved in classification of environmental information processing, metabolism, and cellular processes were identified. **(B)** Of the biologic procession, the significantly enriched terms were identified in Figure 4B. **(C)** Metabolic pathway enrichment was detected based on metabolite set enrichment analysis. **(D)** Overlap between the gene list involved in butanoate metabolism and DEGs of GSE132176.

KEGG pathway analysis revealed that DEMs associated with chronic hypoxia-induced ToF-related myocardial pathogenesis were mainly enriched in the AMPK signaling pathway (cluster frequency = 5.88%, *p* = 1.80E−03), aldosterone synthesis and secretion (cluster frequency = 5.88%, *p* = 5.80E−03), purine metabolism (cluster frequency = 11.76%, *p* = 5.80E−03), longevity regulating pathway (cluster frequency = 3.92%, *p* = 9.93E−03), and cGMP–PKG signaling pathway (cluster frequency = 3.92%, *p* = 9.93E−03) (Figures 5A,B; Supplementary Table 5).

MSEA revealed the enrichment of the butanoate metabolism (*p* = 0.0029; hit compound: 2-hydroxyglutarate), purine metabolism (*p* = 0.0029; xanthine, L-glutamine, ADP, adenosine, xanthosine, IDP, hypoxanthine, inosine, guanine, deoxyguanosine, guanosine, adenine, and urea), pantothenate and CoA biosynthesis (*p* = 0.0029; pantetheine, L-valine, L-cysteine, and uracil), fructose and mannose metabolism (*p* = 0.0029; D-fructose and D-mannose), and starch and sucrose metabolism (*p* = 0.0029; D-fructose and D-glucose) pathways (Figure 5C; Supplementary Table 5).

### Detecting Butanoate Metabolism–Related Regulators

**Figure 6:**
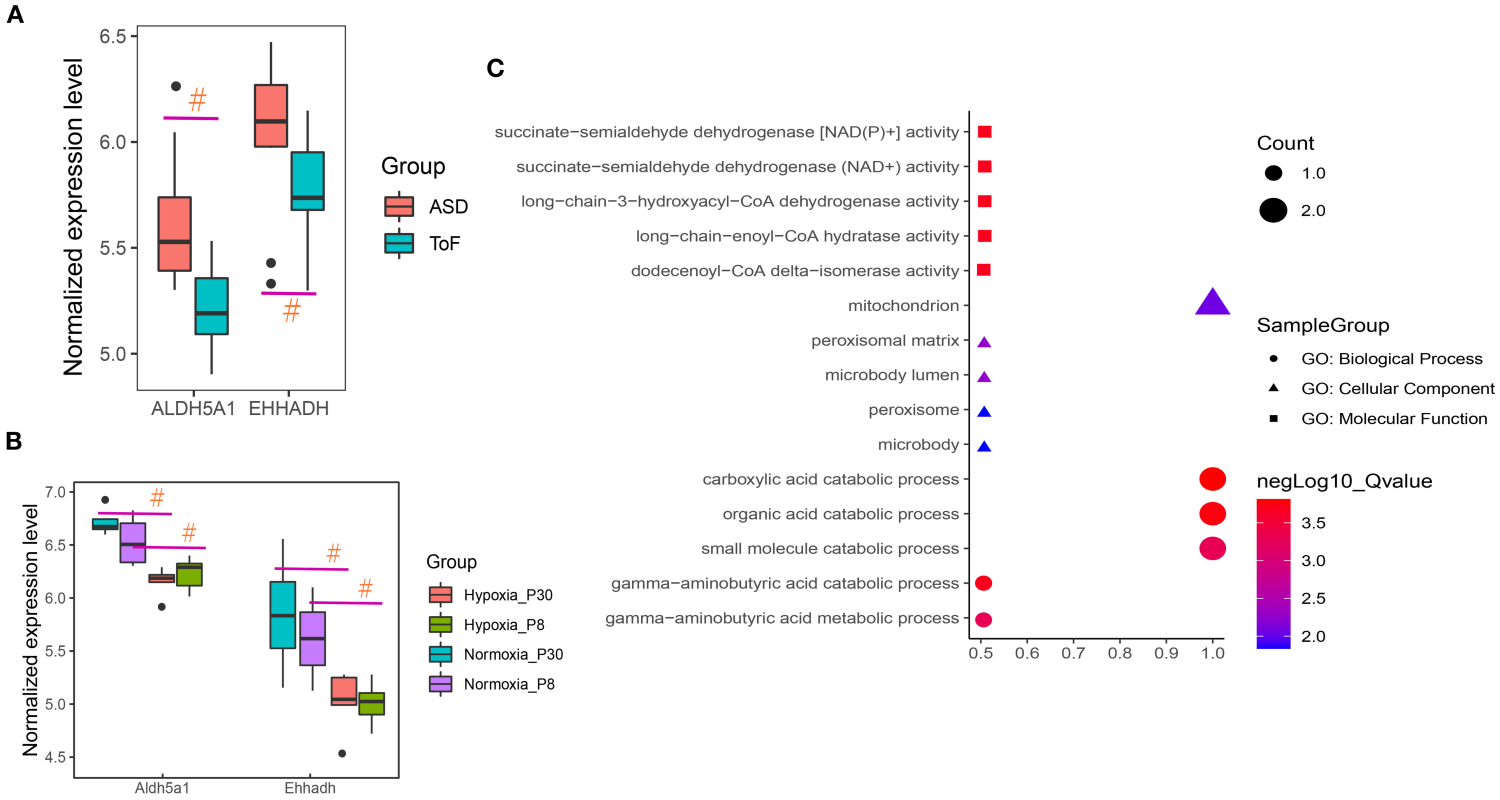
Hub regulator expression and functional enrichment analysis of ToF and ASD RA specimens. **(A,B)** Hub genes showed differences in ToF and ASD RA specimens in the GSE132176 and GSE169214 datasets, respectively. **(C)** Hub terms were enriched in response to hub regulators. ^#^*p* < 0.05.

After overlap, the hub regulators 3-hydroxyacyl-CoA dehydrogenase (EHHADH) and aldehyde dehydrogenase 5 family member A1 (ALDH5A1) were detected in response to transcriptome DEGs and butanoate metabolism pathway (Figure 5D). In addition, ALDH5A1 and EHHADH expression levels were significantly downregulated in ToF samples in GSE132176, as the cyanotic congenital heart tissues in GSE169214 (Figures 6A,B).

Functional enrichment analyses revealed the involvement of carboxylic acid catabolic (*p* = 1.54E−04; gene list: EHHADH and ALDH5A1), organic acid catabolic (*p* = 1.73E−04; EHHADH and ALDH5A1), and gamma-aminobutyric acid catabolic (*p* = 1.98E−04; ALDH5A1) processes (Figure 6C).

## Discussion

In this preliminary study, we used microarrays to analyze ToF and ASD RA specimens to explore changes in the transcriptomic profile and metabolic pathways, and mapped a metabolic pathway regulatory network for chronic hypoxia in ToF (4, 7). We also performed metabolomic profiling to study changes in metabolites in myocardial tissues, assess the association between these changes and ToF progression, establish metabolic profiles of ToF, and analyze metabolomic changes in patients with ToF, with the aim of providing novel insights into the etiology and pathogenesis of ToF.

Transcriptomic and metabolomic analyses suggested that metabolic reprogramming is involved in the process of myocardial damage and remodeling in ToF. At the gene expression level, the metabolic pathway changes mainly involved regulation of lipase activity, regulation of phospholipase activity, and regulation of protein metabolic process (11). The genes encoding EHHADH and ALDH5A1 showed differences in the pathological progression of ToF. Through metabolomic profiling, we identified 49 up- and 24 downregulated DEMs from a total of 1,402 metabolites; of them, adenosine 5′-monophosphate, 2′-deoxyguanosine-5′-monophosphate, guanosine-5′-monophosphate, xanthosine-5′-monophosphate, and 3′-adenylic acid were found to be significantly upregulated, while TG (8:0_18:1_18:2), 2-methylsuccinic acid, glutaric acid, ethylmalonate, and dimethylmalonic acid were significantly downregulated. Furthermore, metabolic pathway enrichment analysis indicated that butanoate and purine metabolism might be involved in the pathological progression of ToF at the metabolite level.

Butanoate, a short-chain fatty acid, is closely associated with cell proliferation, apoptosis, and carcinogenesis. It can be used to regulate or treat intestinal flora imbalance, enteritis, diarrhea, and other diseases. In the cardiovascular field, industry is closely related to myocardial infarction, hypertension, and the risk of cardiovascular disease (28, 29). Based on the PROMINENT study, Pradhan et al. reported that butanoate metabolism dysfunction, which is involved in lipid peroxidation, is significantly correlated with myocardial infarction, stroke, and coronary revascularization (30). In addition, in patients with type II diabetes, Araki et al. reported a significant correlation between lipid and butanoate metabolism markers and pathological changes involved in vascular sclerosis, inflammation, and abnormal lipid deposition (31). Walejko et al. identified that in the newborn heart, DEMs were significantly related to lipid metabolism, fatty acid function, and mitochondrial oxidative phosphorylation, which provides a basic understanding of the mechanism underlying cardiogenesis, fibrosis remodeling, and heart failure (32).

Xu et al. found that the high expression level of the gene encoding EHHADH may be closely related to heart damage caused by exposure to hexafluoropropylene oxide dimer acid (33). Gholaminejad et al. suggested that the gene encoding EHHADH plays an important role in the development of diabetic nephropathy in end-stage renal disease (34). In addition, Chen et al. illustrated that EHHADH expression was significantly upregulated in the left atrial biopsy specimens of patients with mitral regurgitation compared with those with aortic valve disease (35). Niimi et al. suggested that ALDH5A1 expression is significantly correlated with the pathogenesis of diabetic neuropathy, a peripheral nervous system disorder (36). Gibson et al. indicated that the protease of succinate semialdehyde dehydrogenase, coded by Aldh5a1, seems to play a chief role in regulating myocardial metabolism and energy balance (37). Fuentealba et al. found that ALDH5A1 expression is upregulated in long-lived mouse models of aging as compared with that in short-lived models, suggesting that ALDH5A1 is a hub regulator and that it might be closely related to life- and healthspan (38) (Figure 6).

This study is of certain research significance considering that it involves transcriptomic and metabolomic analyses of clinical samples. However, limited by sample size and long-term follow-up data, we cannot explain the translational importance of relevant core metabolites for prognosis prediction or clinical treatment of ToF. Another limitation is the discussion of pertinent molecular mechanisms. Validation based on *in vivo* and *in vitro* experiments is necessary. In the future, our aim is to increase sample size and conduct a prospective clinical follow-up study to explore metabolites of significant clinical translational value.

## Conclusions

Through public database-based transcriptomic and metabolomic analyses of clinical samples, we elucidated the pathological development of ToF at the gene and metabolite level, revealing the potential role of cardiac metabolic reprogramming in cyanotic congenital heart disease.

## Data Availability Statement

The datasets presented in this study can be found in online repositories. The names of the repository/repositories and accession number(s) can be found in the article/Supplementary Material.

## Ethics Statement

This study approved by the Institute of Ethics Committee of Henan people's Hospital and Zhengzhou University (Acceptance number: G2021-107). Written informed consent to participate in this study was provided by the participants' legal guardian/next of kin.

## Author Contributions

JL and SK drafted the article, were accountable for all aspects related to reliability, and freedom from bias of the data presented herein and their interpretation. SS and HD were involved in statistical analyses and data interpretation. ZZ and TF provided guidance, evaluated the entire article, and approving the final submitted version. All authors have read and approved the final article.

## Funding

This study was funded by the Medical Science and Technology Research Project of Henan Province of China (SBGJ202001005) and Medical Science and Technology Research Project of Henan Province of China (201502021).

## Conflict of Interest

The authors declare that the research was conducted in the absence of any commercial or financial relationships that could be construed as a potential conflict of interest.

## Publisher's Note

All claims expressed in this article are solely those of the authors and do not necessarily represent those of their affiliated organizations, or those of the publisher, the editors and the reviewers. Any product that may be evaluated in this article, or claim that may be made by its manufacturer, is not guaranteed or endorsed by the publisher.

## Supplementary Material

Supplementary Table 1 Differentially expressed genes and functional enrichment analysis results pertaining to ToF-related myocardial damage.

Supplementary Table 2 Gene set enrichment analysis.

Supplementary Table 3 Gene set variation analysis of metabolic pathway score in response to ToF-related metabolic reprogramming.

Supplementary Table 4 Differentially expressed metabolites in right atrial biopsies.

Supplementary Table 5 KEGG and metabolite set enrichment analysis of ToF-related cardiac DEMs.
